# An assessment of the prevalence of cannabis use in eye clinic patients and its implications on glaucoma diagnosis and management

**DOI:** 10.1007/s10792-025-03846-2

**Published:** 2025-11-16

**Authors:** Andrew J. Adamek, Musse A. Hussein, Iya Abdulkarim, Silvia Orengo-Nania, Huda Sheheitli

**Affiliations:** 1 University of Minnesota College of Biological Sciences, Minneapolis, Minnesota USA; 2 Department of Ophthalmology and Visual Neurosciences, University of Minnesota, Phillips Wangesteen Building, 9th floor, 516 Delaware St SE, Minneapolis, Minnesota 55455 USA; 3 University of Minnesota Medical School, 420 Delaware St SE, Minneapolis, Minnesota 55455 USA

**Keywords:** Glaucoma, Cannabis, Screening, Intraocular pressure, Cannabinoid

## Abstract

**Purpose:**

Intraocular pressure (IOP) is a major clinical marker used to diagnose glaucoma and monitor its treatment. Marijuana use can confound IOP measurements by temporarily lowering the IOP, potentially leading to missed diagnoses of glaucoma or a false sense of effective treatment. Therefore, factors that may affect diagnosis or treatment of glaucoma need to be characterized and considered. We aim to assess the prevalence of cannabis use among patients presenting for an eye examination.

**Methods:**

A survey was administered to 134 patients arriving for their regular ophthalmic appointments, from Oct 2022 to Jan 2023 and Jul 2024–Aug 2024 in four eye clinics at the University of Minnesota (UMN).

**Results:**

Among study patients, 15.7% reported recent use of marijuana (< 1 month), 8.2% described themselves as regular users, and 4.5% of patients reported using it every day. Just under half (44.2%) of glaucoma patients indicated they were interested in using marijuana for their glaucoma. Patients that used marijuana less than 24 h from their eye exam were significantly more likely to know that marijuana decreases intraocular pressure (IOP) (*p* = 0.02). Beliefs that marijuana is effective (*p* = 0.016), lowers IOP (*p* = 0.011), and has fewer side effects than glaucoma medications (*p* = 0.014) were predictive of interest in marijuana use. In contrast, beliefs that marijuana has negative physical (*p* = 0.041) and mental (*p* = 0.003) health effects were predictive of disinterest in use.

**Conclusion:**

These findings indicate need for increased patient screening for recent marijuana use so ophthalmologists may contextually assess IOP and educate patients on marijuana’s effects on the diagnosis and treatment of glaucoma.

**Supplementary Information:**

The online version contains supplementary material available at 10.1007/s10792-025-03846-2.

## Introduction

Glaucoma is an asymptomatic disease in early stages that slowly progresses; therefore, it is often underdiagnosed. It is estimated that between 10 and 33% of patients had advanced disease and visual impairment at their first diagnosis because of the late presentation of the disease [1–3]. Furthermore, approximately 2.4 million Americans, or around half of individuals with glaucoma are undiagnosed [4, 5], and an estimated 78% of those with glaucoma have been previously undiagnosed and untreated [4]. This is especially concerning considering that up to 40% of retinal ganglion cells can be destroyed before visual field defects are noticed [6]. Thus, early detection and screening is vitally important in this disease [7].

Accurate measurement of IOP is one of the major tools used by eye care providers to screen patients for glaucoma in a clinic setting [8]. Elevated intraocular pressure is a major risk factor for glaucoma and is the only known modifiable risk factor that can impact disease outcome [9]. Numerous pharmacologic agents have been shown to reduce intraocular pressure in patients including, carbonic anhydrase inhibitors, cholinergic agents, statins, beta blockers, and marijuana [10].

One of the first studies to quantify effects of marijuana on IOP was an observational study of healthy patients in 1971. This study revealed a range from 15% (5 mg ingested at 180 min) to 34% reduction of IOP (4% THC smoked measured at 30 min) after use of marijuana [11]. This effect has been corroborated in more recent double-blind, though small, RCTs for healthy and glaucoma patients through inhaled, ingested, and sublingual administration of THC for 3–5 h [12–14]. Examination of plasma THC finds a dose dependent relationship up to 20 mg plasma THC, and a peak effect of − 16% IOP at 60 min gradually decreasing to − 7% at 5 h [12]. However, some recent studies, including a phase 2 clinical trial of topical administration of THC, have shown some ineffectiveness of THC’s ability to lower IOP [15, 16].

While the exact mechanism of IOP-lowering action of cannabinoids is still under research, the current hypotheses for cannabinoids’ method of action include activation of cannabinoid 1 (CB1), G protein-coupled receptor 18 (GPR18), and G protein-coupled receptor 119 (GPR119) receptors [17–20]. In vitro binding assays have shown that the THC isomer, trans delta-9-tetrahydrocannabinol (delta-9-THC) and cannabigerol bind to these CB1 receptors [20, 21]. These compounds are found in high concentrations in several different tissues in the eye, specifically in Schlemm’s canal, shown in ex vivo human tissue and murine samples [17–19]. The function of this binding is outside the scope of this paper and reviewed elsewhere [20, 22, 23]; briefly, this binding may function to decrease aqueous humor production, shown in monkeys [24], and/or increasing aqueous outflow, as suggested by Porcella et al. in their work on murine samples [25].

Although cannabis has demonstrated IOP-lowering effects, research has not shown it to be a definitively effective treatment for glaucoma. The current statement by the American Academy of Ophthalmology (AAO) is that there is “no scientific evidence demonstrating increased benefit and/or diminished risk of marijuana use in the treatment of glaucoma compared with the wide variety of pharmaceutical agents now available” [26]. This is due to several reasons; First, as previously discussed, the effects of marijuana on IOP are transient (3–5 h). Because glaucoma is a condition that needs continual therapy, dosing 5–8 times a day with an estimated 18–20 mg per dose would be impractical for patients [13, 26]. In addition, because no effective topical formulation of THC has been developed, the effects of marijuana therapy would be systemic—for example, postural hypotension, palpitations, and impairing effects on the brain—which would need to be present 24/7 for glaucoma management [13, 26]. Finally, the AAO warns that marijuana may be damaging to the optic nerve given the systemic hypotensive effects that may reduce blood flow to this structure [26]. Nevertheless, marijuana remains medically accessible as a treatment option for glaucoma.

At the time of writing, marijuana use for recreational purposes is legal in 24 states as well as the District of Columbia (D.C.) and it is legal for medical purposes in 40 states and D.C [27]. This is a relatively recent event in the United States, with the first states legalizing marijuana for recreational use in 2012 and the first medical legalization in 1996 [28]. In Minnesota, where this study took place, medical marijuana use, including for glaucoma, has been legal since 2014, and recreational use for low-dose, hemp-derived cannabis edibles for adults since 2022 [29]. With these recent legalizations, the prevalence of marijuana use for recreational and medical purposes has been on the rise. Between 2002 and 2019 in the U.S., the prevalence of marijuana use rose from 10.4 to 18.0% for past-year users and from 1.3 to 3.9% for individuals who used it more than 300 days annually [30]. The prevalence of medical cannabis use is estimated to be around 2–2.5% of U.S. adults [31, 32].

Given the significant increase in marijuana use across the United States, it is important to obtain more information regarding how this may impact ophthalmic clinic visits. Understanding the frequency of marijuana use in ophthalmic patients (both glaucomatous and non-glaucomatous) is crucial because marijuana administration around the time of ophthalmic evaluation can potentially temporarily impact IOP measurements and play a role in inaccurate assessment of the patient’s IOP. As a result, these inaccurate IOP measurements could alter both the management and diagnosis of glaucomatous disease.

An extensive literature review was performed which demonstrated that there are only a few studies that have examined the prevalence of marijuana use in glaucoma patients, with estimates ranging from 2.6 to 4.4% [33–35]. Not only was there a scarcity of work on the prevalence of marijuana use in glaucoma patients, but there was also a significant lack of published data examining prevalence of marijuana use in non-glaucoma patients presenting to the eye clinic. Furthermore, these articles only characterize the prevalence of marijuana use for glaucoma treatment. It is also vital to consider that patients also use marijuana for other uses, for example recreationally or for pain, that can affect glaucoma diagnosis and management. Thus far, to our knowledge, the prevalence of generalized marijuana use has not been characterized for eye clinic patients. Most glaringly, there was not any substantial work specifically examining temporality (i.e. recent use of marijuana in relation to the time of an eye clinic appointment and IOP testing). Recent use is specifically critical in understanding how many patients may be at risk of having artificially reduced IOP at their eye exam, which can impact glaucoma diagnosis and treatment.

Our study aimed to fill this knowledge gap. We had two primary aims: 1) to determine the prevalence of marijuana use in both glaucoma and non-glaucoma patients who present for eye exams, which include IOP measurements; 2) to determine what factors are associated with current usage and desire to use in the future. These aims will aid in assessing the necessity for providers to screen for marijuana use in the eye clinic setting. To do this, we conducted a cross-sectional prevalence survey administered to patients during their eye clinic visit at the University of Minnesota.

## Methods

### Overview

A cross-sectional survey study was conducted from October 2022 to January 2023 and July 2024 to August 2024 in four different eye clinics at the University of Minnesota (UMN). The study was approved by the UMN Institutional Review Board (IRB) (STUDY00015654) and all associated ancillary reviews including Oncore, Fairview, and the UMN Health Information Privacy & Compliance Office (HIPCO). All patients gave informed written consent and written authorization of patient information access in accordance with the Health Insurance Portability and Accountability Act and the UMN HIPCO. Study team members gave assurances to the patients that the information would remain confidential, that no legal action could result from this study, and that the clinical care would not change by agreeing or disagreeing to take place in the study.

### Participants

Clinic patients were asked to participate in a short survey while they were waiting to be seen by their doctor as long as they did not meet the exclusion criteria of certain categories of vulnerable populations according to the UMN IRB protocol (i.e., children, pregnant women, prisoners, and adults with absent or diminished capacity to consent) [36]. Patients were not excluded based on their ocular history: individuals with and without a known diagnosis of glaucoma were included in the study to ensure a representative sample. If the patient agreed to participate in the study, a study team member briefed the patient on the study and then obtained informed written consent from the patient.

### Survey

After consent, the survey was either administered to the patient by a study team member or the patient completed a paper copy of the survey in the private setting of the exam room. The first part of the survey assessed demographic information (e.g., ethnicity, income, education level, marital status) (Online Resource 1). The second part of the survey assessed current knowledge about marijuana use in glaucoma, marijuana use in family and friends, and about past and current usage of marijuana for the patient (Online Resource 1). The final part of the survey was adapted from Belyea et al. who developed a questionnaire to evaluate patient perceptions of marijuana and glaucoma as well as predicting intentions to use marijuana for glaucoma [34] (Online Resource 2). This questionnaire is a scale from “strongly agree” to “strongly disagree” of various statements regarding marijuana and glaucoma. The patients were informed that not knowing or having an opinion was acceptable and that any “unknown” responses were recorded as such. These questions were used to assess patients’ desire to use marijuana, their knowledge of marijuana in the context of glaucoma, and factors that may be correlated to current use or desire to use. Relevant medical information including medical record number, age, sex, glaucoma diagnosis, severity of glaucoma, glaucoma treatment, and glaucoma surgeries were collected form the patient’s medical chart.

### Data analysis

All data was securely collected on REDCap (Research Electronic Data Capture) hosted at UMN [37, 38], exported to Excel, and then stored in UMN Box storage. Participant demographics, clinical characteristics, and survey responses were summarized as means/standard deviations (SD) and median/interquartile range (IQR) for continuous variables, and frequencies and percentages for categorical variables. Associations between responses were assessed using either Pearson’s χ^2^ test or Fisher’s Exact test for categorical outcomes as appropriate. Bivariate logistic regressions were calculated using Firth’s method for selected variables—patients who responded “unsure” to either the independent or dependent variables were excluded for each analysis. U.S. estimates were compared to this study using a two-sample proportion test. All analyses were conducted at the 0.05 significance level using the R statistical software (version 4.2.0) and JMP Pro statistical software (18.0.02).

## Results

### Demographics

152 patients were asked to participate in the study—18 declined, resulting in 134 responses (88.2% response rate). Of these patients, 113 (84.3%) were being managed for glaucoma. The study population was predominantly elderly: 67 (50%) of the patients were 65 years of age or greater. Among participants, 44 (32.8%) had a graduate degree and 80 (59.7%) had a bachelor’s degree or higher (Table 1).

**Table 1:** Baseline characteristics of study patients

Characteristics	Total (n = 134)
*Age (yrs)*	
Mean (SD)	63.0 (15.9)
Median (IQR)	66 (55, 74)
*Sex, no. (%)*	
Female	62 (46.3%)
Male	71 (53.0%)
Decline to answer	1 (0.7%)
*Race, no. (%)*	
Asian	9 (6.7%)
Black	17 (12.7%)
Native Hawaiian/Pacific Islander	1 (0.7%)
Hispanic/Latino	3 (2.2%)
Middle Eastern	3 (2.2%)
White	92 (68.7%)
More than one race	8 (6.0%)
Decline to answer	1 (0.7%)
*Education level, no. (%)*	
Below high school	9 (6.7%)
Completed high school/GED	14 (10.4%)
Some college	22 (16.4%)
Associate degree	9 (6.7%)
Bachelor’s degree	31 (23.1%)
Some graduate studies	5 (3.7%)
Graduate degree completed	44 (32.8%)
*Clinical glaucoma diagnosis*	
Yes	113 (83.6%)
No	21 (16.4%)
*Clinical glaucoma severity (of those managed for glaucoma)*	
Suspect/mild/moderate	66 (58.4%)
Severe	46 (40.7%)
Unknown	1 (0.9%)

### Prevalence of marijuana usage

Just under half (48.5%, 65/134) of the study population reported having used marijuana at least once in their life, 46.3% (62/134) reported having used it recreationally, 10.4% (14/134) used it medically for conditions other than glaucoma (pain, anxiety, depression, sleep), and 2.2% (3/134) used it medically for glaucoma (Table 2). These categories were not mutually exclusive, as 9.0% (12/134) reported a combination of recreational and medical use. In our study population, 15.7% (21/134) were recent users (as defined as use < 1 month from the clinic visit in which the survey was administered), 8.2% (11/134) were self-reported regular users of marijuana, 4.5% (6/134) reported using marijuana “every day or a few times a day,” and 4.5% (6/134) used it within 24 h before the exam (Table 2). The closest usages of marijuana to the exam were six and 13 h before the exam.

**Table 2:** Marijuana use prevalence for all patients and by glaucoma status

Characteristics	All patients (n = 134)	Patients managed for glaucoma (n = 112)	Other patients (n = 22)	*p*-value
Lifetime marijuana use	65 (48.5%)	51 (45.5%)	14 (63.6%)	0.12
Lifetime recreational use	62 (46.3%)	49 (43.8%)	13 (59.1%)	0.19
Lifetime medical use for glaucoma	3 (2.2%)	3 (2.7%)	NA	NA
Lifetime medical use for other conditions	14 (10.4%)	8 (7.1%)	6 (27.3%)	** < 0.01**
Self-described regular user (of those who have used marijuana)	11/65 (16.9%)	8/51 (15.7%)	3/14 (21.4%)	0.61
Recreational use only	5/65 (7.7%)	3/51 (5.9%)	1/22 (4.5%)	
Medical and recreational use	6/65 (9.2%)	5/51 (9.8%)	2/22 (9.1%)	
Recent users (< 1 month from clinic visit)	21 (15.7%)	15 (13.4%)	6 (27.3%)	0.10
Non-recent users (> 1 month from clinic visit)	44 (32.8%)	36 (32.1%)	8 (36.4%)	
Never used	69 (51.5%)	61 (54.5%)	8 (36.4%)	
Of users in past 24 h, how long ago was last usage (hours)?	n = 6 (4.5%)	n = 5 (4.5%)	n = 1 (4.5%)	NA
Mean (SD)	14.7 (5.56)	14.1 (6.25)	NA	
Median (IQR)	16 (9.75–19)	14.75 (7.88–19.75)	NA	

Lifetime recreational and medical use is non-exclusive, meaning the total lifetime use may be smaller than the sum of lifetime recreational and medical use. The *p*-value is calculated from the difference in means between glaucoma and non-glaucoma patients. *P*-values below the stated significance level of 0.05 are bold. NA, Not applicable, for example since non-glaucoma patients cannot use marijuana medically for glaucoma

The lifetime prevalence of medical use of marijuana was greater for non-glaucoma patients than those being managed for glaucoma (27.3% vs. 9.8%, Pearson’s χ^2^ test, *p* = 0.02) (Table 2). However, there was no statistically significant difference in the lifetime marijuana use (recreational or medical) (63.6% vs. 45.5%, Pearson’s χ^2^ test, *p* = 0.12) and lifetime recreational use of marijuana (59.1% vs. 43.8%, Pearson’s χ^2^ test, *p* = 0.19) for non-glaucoma and glaucoma patients (Table 2). Additionally, there was no statistically significant difference in number of recent (< 1 month from clinic visit) and non-recent users (> 1 month use) of marijuana for non-glaucoma and glaucoma patients (27.3% vs. 13.4% recent users, Pearson’s χ^2^ test, *p* = 0.10) (Table 2).

Those who used marijuana recently were statistically significantly younger than non-recent users and those who had never used marijuana (Pearson’s χ^2^ test, *p* = 0.02) (Table 3). There were no other significant correlations between demographic factors and recency of use of marijuana (Table 3).

**Table 3:** Marijuana use recency by demographic variables

Demographic	Recent use (< 1 month)	Non-recent use (> 1 month)	Never used	*p*-value
Age				**0.02**
18–34 (n = 9)	4 (44.4%)	1 (11.1%)	4 (44.4%)	
35–65 (n = 57)	9 (15.8%)	24 (42.1%)	24 (42.1%)	
> 65 (n = 67)	5 (7.5%)	23 (34.3%)	39 (58.21%)	
Sex				0.87
Female (n = 62)	8 (12.9%)	21 (33.9%)	33 (53.2%)	
Male (n = 72)	10 (13.9%)	27 (37.5%)	35 (48.6%)	
Education level				
Below high school (n = 9)	2 (22.2%)	1 (11.1%)	6 (66.7%)	0.18
Completed high school/GED (n = 14)	0 (0%)	3 (21.4%)	11 (78.6%)	
Some college (n = 22)	5 (22.7%)	7 (31.8%)	10 (45.5%)	
Associate degree (n = 9)	2 (22.2%)	2 (22.2%)	5 (55.6%)	
Bachelor’s degree (n = 31)	6 (19.4%)	11 (35.5%)	14 (45.2%)	
Some graduate studies (n = 5)	0 (0.0%)	2 (40.0%)	3 (60.0%)	
Graduate degree completed (n = 44)	3 (6.8%)	22 (50.0%)	19 (43.2%)	
Race/Ethnicity				0.45
Asian (n = 9)	0 (0%)	2 (22.2%)	7 (77.8%)	
Black (n = 18)	45 (22.2%)	5 (27.8%)	9 (50.0%)	
Hispanic/Latino (n = 4)	1 (25.0%)	1 (25.0%)	2 (50.0%)	
Middle Eastern (n = 3)	0 (0%)	0 (0%)	3 (100%)	
Native Hawaiian or other Pacific Islander (n = 1)	0 (0%)	0 (0%)	1 (100%)	
White (n = 92)	11 (12.0%)	37 (40.2%)	68 (50.8%)	
Adjusted income level				0.054
(1st quartile) (n = 26)	5 (19.2%)	6 (23.1%)	15 (57.7%)	
(2nd quartile) (n = 25)	1 (4.0%)	11 (44.0%)	13 (52.0%)	
(3rd quartile) (n = 25)	4 (16.0%)	6 (24.0%)	15 (60.0%)	
(4th quartile) (n = 25)	5 (20.0%)	14 (56.0%)	6 (24.0%)	
Glaucoma severity				0.97
Suspect, mild, or moderate (n = 66)	7 (10.6%)	23 (34.9%)	36 (54.6%)	
Severe (n = 46)	5 (10.9%)	17 (37.0%)	24 (52.2%)	

*P*-values were calculated with a Pearson’s χ^2^ test. Annual adjusted income is calculated as $\frac{{House\;hold\;income}}{{(House\;hold\;size)^{{0.5}} }}$. *P*-values represent the difference in percentage for each category of usage among categories (e.g., male vs. female) in each demographic variable. *P*-values below the significance level of 0.05 are bold

### Knowledge of marijuana in glaucoma

Of patients surveyed regarding their knowledge of the effects of marijuana on glaucoma (n = 94), 58.5% (55/94) were aware of marijuana’s use for glaucoma before the survey. Their sources of this information were through media and the news (63.2%, 36/57), followed by interpersonal communication (22.8%, 13/57), personal research (7.0%, 4/57), and medical providers (7.0%, 4/57). The percentage of glaucoma and non-glaucoma patients who were aware of marijuana’s use in glaucoma was similar (glaucoma patients = 42/74, 56.8%; non-glaucoma patients = 13/26, 50%). Additionally, 8.5% (8/94) had family members or friends who have used marijuana for their glaucoma. Of these patients, 71.4% (5/7; 1 patient did not answer) were recommended to start using marijuana for their glaucoma by this family member or friend.

**Fig. 1:**
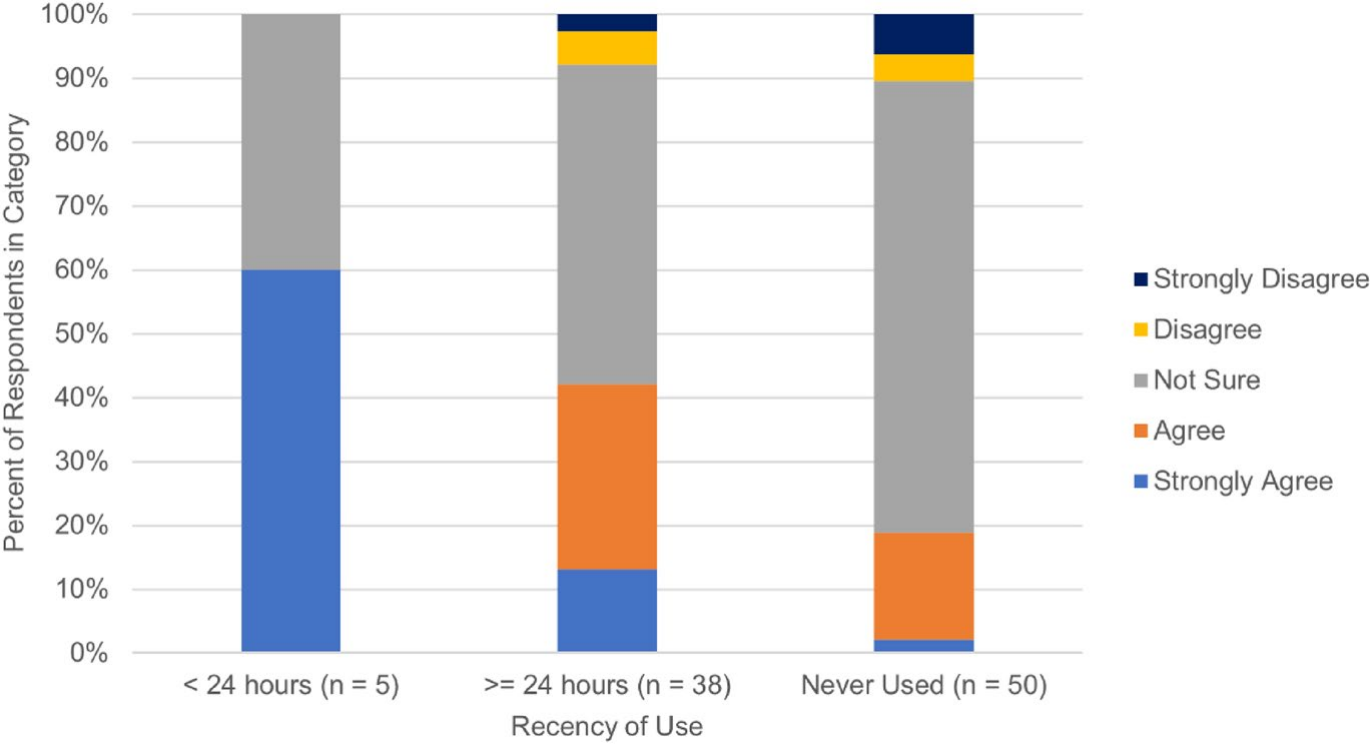
Knowledge of marijuana’s effect on IOP by < 24 h use. A stacked column chart showing percent agreement to question 5, that marijuana can lower IOP, by marijuana use < 24 h and >  = 24 h from the clinic visit and those who reported to have never used marijuana. Those who used marijuana within 24 h of survey administration were significantly more likely to strongly agree that marijuana lowers IOP than the other patient categories (Fischer’s Exact, *p* = 0.02)

Of all surveyed patients, 58.5% (55/94) were not sure or had no opinion if marijuana lowered IOP, 31.6% believed that marijuana lowered IOP, and 8.6% stated they didn’t believe marijuana lowered IOP (Table 4). Additionally, 30.4% (28/92) patients agreed that marijuana was an effective treatment for glaucoma, 5.5% (5/92) disagreed, and 64.1% (59/92) said they were not sure or had no opinion. A significantly greater percentage of non-glaucoma patients agreed that marijuana is an effective treatment for glaucoma (60% vs. 22.2%, Fisher’s Exact test, *p* < 0.01) and that marijuana decreases intraocular pressure (55% vs. 25%, Fisher’s Exact test, *p* = 0.03) when compared to glaucoma patients (Table 4). Of note, those who used marijuana < 24 h from the examination time had a significantly higher level of agreement that marijuana lowered IOP than those who used it > 24 h from the examination time or who had not used marijuana (Fisher’s Exact Test, *p* = 0.02) (Fig. 1).

**Table 4:** Survey responses for knowledge of marijuana in the context of glaucoma

Survey statement	All patients (n = 94)	Patients managed for glaucoma (n = 74)	Other patients (n = 20)	*p*-value
I think marijuana is an effective treatment for glaucoma				** < 0.01**
Agree or strongly agree	28 (30.4%)	16 (22.2%)	12 (60%)	
Disagree or strongly disagree	5 (5.5%)	5 (6.9%)	0 (0%)	
No opinion/not sure	59 (64.1%)	51 (70.8%)	8 (40%)	
Using marijuana can lead to a decrease in intraocular pressure				**0.03**
Agree or strongly agree	29 (31.6%)	18 (25.0%)	11 (55%)	
Disagree or strongly disagree	8 (8.6%)	8 (11.1%)	0 (0%)	
No opinion/not sure	55 (59.8%)	46 (63.9%)	9 (45%)	
The use of marijuana for glaucoma can have negative effects on the heart, lungs, and brain				0.95
Agree or strongly agree	38 (41.3%)	29 (40.3%)	9 (45%)	
Disagree or strongly disagree	20 (21.7%)	20 (27.8%)	4 (20%)	
No opinion/not sure	34 (37%)	23 (31.9%)	7 (35%)	
The use of marijuana for glaucoma can have negative effects on mental health				0.11
Agree or strongly agree	34 (36.9%)	29 (40.3%)	5 (25%)	
Disagree or strongly disagree	31 (31.7%)	20 (27.8%)	11 (55%)	
No opinion/not sure	27 (29.3%)	23 (31.9%)	4 (20%)	

To show overall agreement or disagreement, we combined “agree” and “strongly agree” into one category and similarly with “disagree” and “strongly disagree.” The *p*-value is calculated by comparing the responses for glaucoma patients and non-glaucoma patients. *P*-values below our stated significance level of 0.05 are bold

### Intentions to use marijuana

**Fig. 2:**
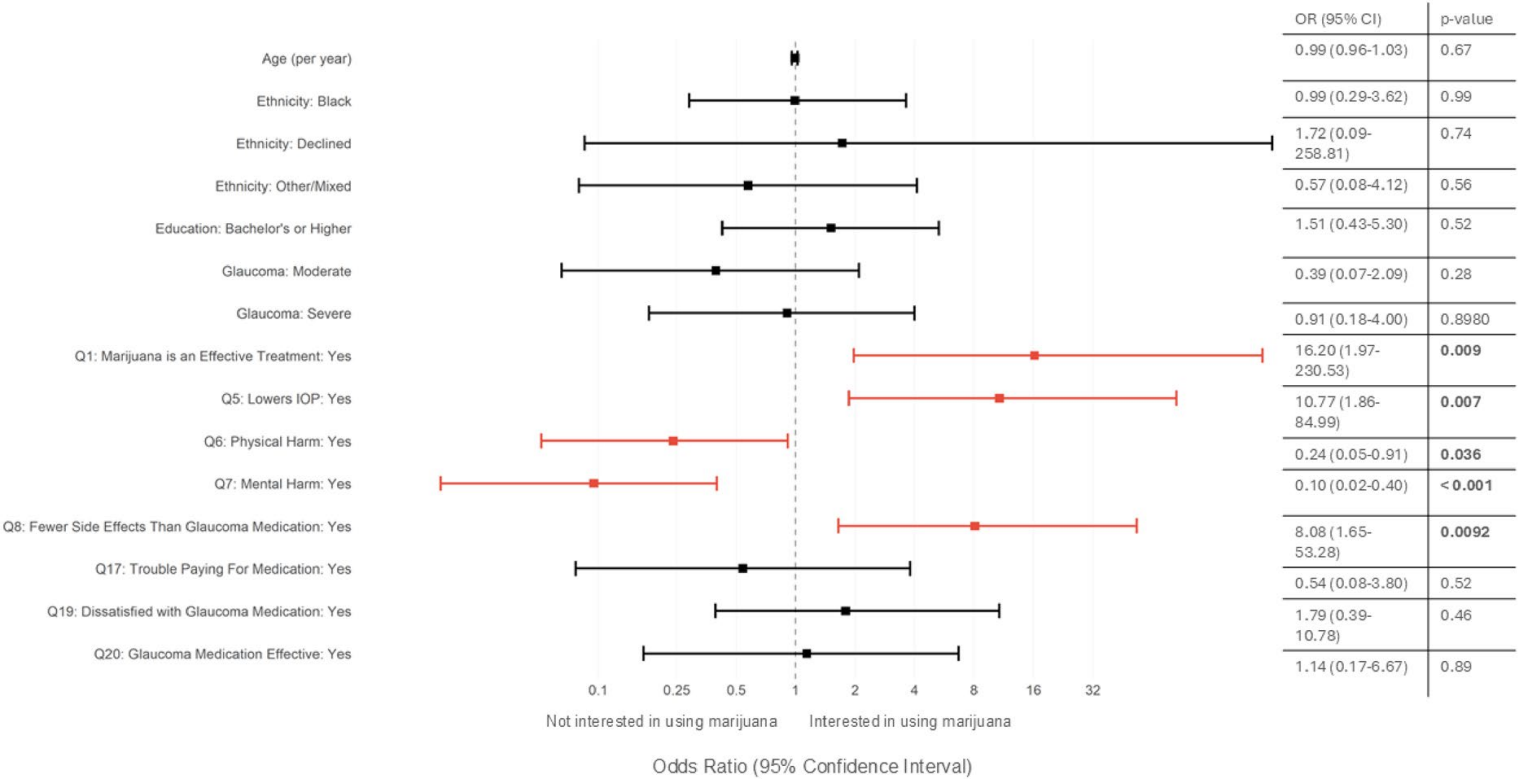
Predictors of Patient Interest in Using Marijuana for Glaucoma. A forest plot showing odds ratios (dots) with 95% confidence intervals (bars) of several tested predictors of patient interest in using marijuana for glaucoma (Question 2). Odds ratios were calculated using Firth’s logistic bivariate regression. Red bars indicate significance (*p* < 0.05). Reference variables were used for multi-level variables: ethnicity (Asian), education (some college/Associate’s), and glaucoma severity (mild), question variables (“disagree or strongly disagree”). Numerical values and *p*-values given on the right

Just under half (44.2%, 50/113) of glaucoma patients were interested in using marijuana for their glaucoma (Table 5). A bivariate logistic regression was run to identify factors that predict a patient’s interest in using cannabis for glaucoma. These results showed that interest in using cannabis is not related to age (OR = 0.99 [0.96–1.03], *p* = 0.67), ethnicity (*p* > 0.56 for all ethnicities), education (*p* > 0.52 for all education levels), or glaucoma severity (*p* > 0.28 for all glaucoma degree categories) (Fig. 2). Instead, patients likely to be interested in using marijuana for their glaucoma were those who agreed that “marijuana is an effective treatment for glaucoma” (Question 1) (Pearson’s χ^2^: *p* < 0.001, Firth’s logistic regression: OR 16.2 [1.97–230.53], *p* = 0.009), that “using marijuana can lead to a decrease in intraocular pressure” (Question 5) (Pearson’s χ^2^: *p* = 0.005; Firth’s logistic ordinal regression: OR 10.77 [1.86–84.99], *p* = 0.007), and that marijuana has fewer side effects than glaucoma medications (Question 8) (Pearson’s χ^2^: *p* < 0.001; Firth’s logistic ordinal regression: OR 8.08 [1.65–53.28], *p* = 0.009) (Fig. 2). However, those that believe that marijuana has negative physical (Question 6) (Pearson’s χ^2^: *p* < 0.001; Firth’s logistic regression: OR: 0.24 [0.05–0.91], *p* = 0.036) and mental health (Question 7) (Pearson’s χ^2^: *p* = 0.004;; Firth’s logistic regression: OR: 0.10 [0.02–0.40], *p* < 0.001) effects were less likely to be interested in using marijuana for their glaucoma (Fig. 2). Additionally, glaucoma patients were more likely not to be interested in using marijuana if it is less effective than medications (70.8% disagree), if it costs more (53.1% disagree), and if it meant that they would need to switch doctors just to be prescribed medical marijuana (68.3% disagree) (Table 5).

**Table 5:** Survey responses for intentions to use marijuana for glaucoma patients

Survey statement	Patients managed for glaucoma (n = 74)
*The use of marijuana for medical purposes is now legal in Minnesota. Knowing this, I would be interested in using marijuana as a treatment for my glaucoma condition*	
Agree or strongly agree	36 (52.2%)
Disagree or strongly disagree	22 (31.9%)
No opinion/not sure	11 (15.9%)
*I would be interested in using marijuana for my glaucoma condition even if it is less effective than my regular glaucoma medications*	
Agree or strongly agree	11 (16.9%)
Disagree or strongly disagree	46 (70.8%)
No opinion/not sure	8 (12.3%)
*I would be interested in using marijuana for my glaucoma condition even if it costs more than my regular glaucoma medications*	
Agree or strongly agree	18 (28.1%)
Disagree or strongly disagree	34 (53.1%)
No opinion/not sure	12 (18.8%)
*If my doctor won’t prescribe medical marijuana for my glaucoma, I will seek other doctors who will*	
Agree or strongly agree	15 (23.8%)
Disagree or strongly disagree	43 (68.3%)
No opinion/not sure	5 (7.9%)

To show overall agreement or disagreement, we combined “agree” and “strongly agree” into one category and similarly with “disagree” and “strongly disagree.”

## Discussion

Our objective was to determine the prevalence of recent use of marijuana in eye clinic patients to gauge the need for routine marijuana use screening in eye clinics. Our results are consistent with past literature on the prevalence of marijuana use for glaucoma (0–4.4%) [33–35]. We also show that a clinically significant proportion of patients in our study used marijuana in the past month (15.7%) and daily (4.5%). If our sample of patients is representative of those seeking eye care in the U.S (87.9 million to 99.5 million) [39], our estimates equate to approximately 3.9–4.5 million people who visit the eye doctor every year using marijuana daily.

Furthermore, while marijuana usage in our study population was similar to the usage in the overall U.S. population for every-day use and past-year use (our study vs. U.S. population: 4.5% vs. 3.9% every-day use; 17.9% vs. 18.0% past-year use) [30], our study population had a statistically significantly higher past-month use than the U.S. average (our study vs. U.S. population: 13.4% vs. 6.54%, two-proportion z-test, *p* = 0.001) (Table 6) [30]. This higher rate of use is particularly notable because the study population was predominantly elderly, and historically marijuana use typically decreases with age [40]. In fact, in our study, there was a significantly higher rate of past-month use among all ages groups, including those above 65 years of age compared to the U.S. population (7.5% vs. 2.30%, two-proportion z-test, *p* = 0.005) (Table 6).

**Table 6:** Comparison of frequency of recent use (< 1 month from clinic visit) by age range between the present study population and the U.S. Population

Age range (years old)	Study population	Estimate of U.S. population[30]	*p*-value
Total	13.4% (18/134)	6.54% (25,310/387,157)	**0.001**
18–34	40.0% (4/10)	15.8% (9167/58,009)	**0.038**
35–64	15.8% (9/57)	6.89% (12,872/186,923)	**0.008**
> 65	7.5% (5/67)	2.30% (3271/142,225)	**0.005**

*P*-values are calculated by the difference in proportion (z-test) between our study population and the estimate of the U.S. population. *P*-values below our stated significance level of 0.05 are bold

In this study, the closest use of marijuana to the exam was around six hours. Therefore, none of the 134 patients used marijuana less than 3–5 h from the eye exam, which would directly affect their IOP. However, it is quite reasonable to predict that in a greater sample size, for those that use marijuana daily or in the past month, they may use marijuana before their exam visit in the future.

To our knowledge, this study is also the first to determine the prevalence of marijuana use and perceptions of marijuana in non-glaucoma eye-clinic patients. It is critically important to understand the use and perceptions of marijuana in this population because this is the population that would be affected by failed glaucoma screening impacting diagnosis due to artificially low IOP. The non-glaucoma patients had similar rates of marijuana use (Table 2) and were more likely to agree that marijuana is an effective treatment for glaucoma (Table 4). Given this subgroup’s more favorable sentiments towards marijuana use, they would especially benefit from marijuana use screening prior to their eye exam. More research should be conducted in this domain to corroborate this data.

These findings support the recommendation to implement a screening question, about current marijuana use, during glaucoma IOP screenings and in glaucoma management. Identifying marijuana use poses minimal risk to the patient and could identify populations that may have elevated IOP readings potentially missed due to cannabis use. This study underscores that the size of this population is significant enough to warrant concern, as missed diagnoses or delayed treatment can impact outcomes. Early and effective treatment is crucial for improving the prognosis of glaucoma [41, 42].

This study also highlights the importance of patient education, specifically as it pertains to national ophthalmic recommendations on usage of cannabis in the context of glaucoma and its effects. While most patients had heard of marijuana’s use in glaucoma and a large portion were interested in trying it, a relatively high percentage of patients reported not being sure if marijuana was an effective treatment for glaucoma. Furthermore, patients that used marijuana within 24 h of their clinic visit were significantly more likely to agree that marijuana can lower IOP—indicating incomplete knowledge of the challenges associated with using marijuana before an eye exam. This need for additional education in the clinical setting is important as a vast majority of patients are receiving information about marijuana in the context of glaucoma from the media or family and friends. Additionally, education seems effective in influencing patient behaviors based on our study: those who believed marijuana had negative physical and mental health effects were less likely to be interested, and in addition a majority of patients reported that they would not be interested in using marijuana for their glaucoma if they knew it was less effective than other glaucoma treatment options. Finally, patients say that they would stay with their doctor even if they would not prescribe them marijuana—indicating that physicians should feel empowered to have these conversations about marijuana use, which may be extremely valuable for the patient’s health. These findings are corroborated by Belyea and colleagues who found that perceptions of legality and acceptability, false beliefs about marijuana use for glaucoma treatment, satisfaction with current glaucoma management, and relevance of costs of treatment all had a significant impact on glaucoma patients’ desire to use marijuana [34]. Importantly, our aim, as a result of these findings, is not to necessarily affect marijuana usage for patients with glaucoma, but primarily it is to increase the knowledge that marijuana use may influence diagnosis and treatment of glaucoma, and that patients should be educated about the effects of marijuana on their eye health.

There are several limitations to this study. Future studies should consider using a larger sample size to increase the confidence and statistical power of the results. Our study population is quite representative of the U.S. population by age, gender, and similar daily and yearly marijuana use to the U.S. population, which adds reliability to our results [30, 43]. However, since one of our study objectives was to assess the potential risk of missed glaucoma diagnoses in patients with regular marijuana use, increasing the number of participants is essential to ensure that the observed prevalence accurately represents this population.

Additionally, because marijuana use is still somewhat of a stigmatized behavior—though at the time of writing marijuana use in Minnesota is legal medically and recreationally[29], with restrictions—some patients may have felt uncomfortable answering some of these questions. This could have resulted in a possible underestimation of marijuana use. To confront this, before the study, we reminded patients of the legality of marijuana, the confidentiality of the responses, that these results would not be entered in patients record, and only the study team would have access to the deidentified data. This is a key consideration for the implementation of such a screening question. As marijuana is currently a federal Schedule I drug, patients may be averse to reporting use even in a state with legal marijuana use, especially if their response will be documented. While the Health Insurance Portability and Accountability Act (HIPAA) protects patient health information, it is paramount that providers still discuss with patients the rationale for documenting marijuana use and emphasize that their responses will be used for the sole purpose of optimizing their medical care [44].

In conclusion, this study provides important and novel data on the prevalence of recent marijuana use in eye clinic patients, as well as perceptions towards marijuana in both patients with and without glaucomatous disease. Furthermore, it also establishes and extends evidence for the need of increased education on marijuana in the context of glaucoma to limit usage before eye exam visits [34]. Thus, we suggest that there is a viable case to be made for the use of a screening question in eye clinics about recent marijuana use given its low-cost and ability to stratify a high-risk population. Understanding factors that may contribute to missed or inadequately treated glaucoma is an area of study that needs to be expanded on given the severe and permanent complications that may arise from untreated glaucoma [4, 5]. We hope our study aides in the contextualization of social factors, such as marijuana use, that may impact diagnosis and treatment of glaucoma.

## Supplementary Information

Below is the link to the electronic supplementary material.

Supplementary file1 (DOCX 1.58 MB)

Supplementary file1 (DOCX 16 kb)

## Data Availability

All pertinent data is provided within the manuscript or supplementary information files.

## Abbreviations

AAO: American Academy of Ophthalmology
CB1: Cannabinoid 1
COPD: Chronic obstructive pulmonary disease
delta-9-THC: Delta-9-tetrahydrocannabinol
D.C.: District of Columbia
GPR18: G protein-coupled receptor 18
GPR119: G protein-coupled receptor 119
HIPCO: Health Information Privacy & Compliance Office
IRB: Institutional Review Board
IOP: Intraocular pressure
U.S.: United States
UMN: University of Minnesota
