# Supplementary figures and images for: An assessment of the prevalence of cannabis use in eye clinic patients and its implications on glaucoma diagnosis and management
Source: Int Ophthalmol. 2025 Nov 16;45(1):484. doi: 10.1007/s10792-025-03846-2 (PMC12620322; doi:10.1007/s10792-025-03846-2)

**Online resource 1.** Patient Survey
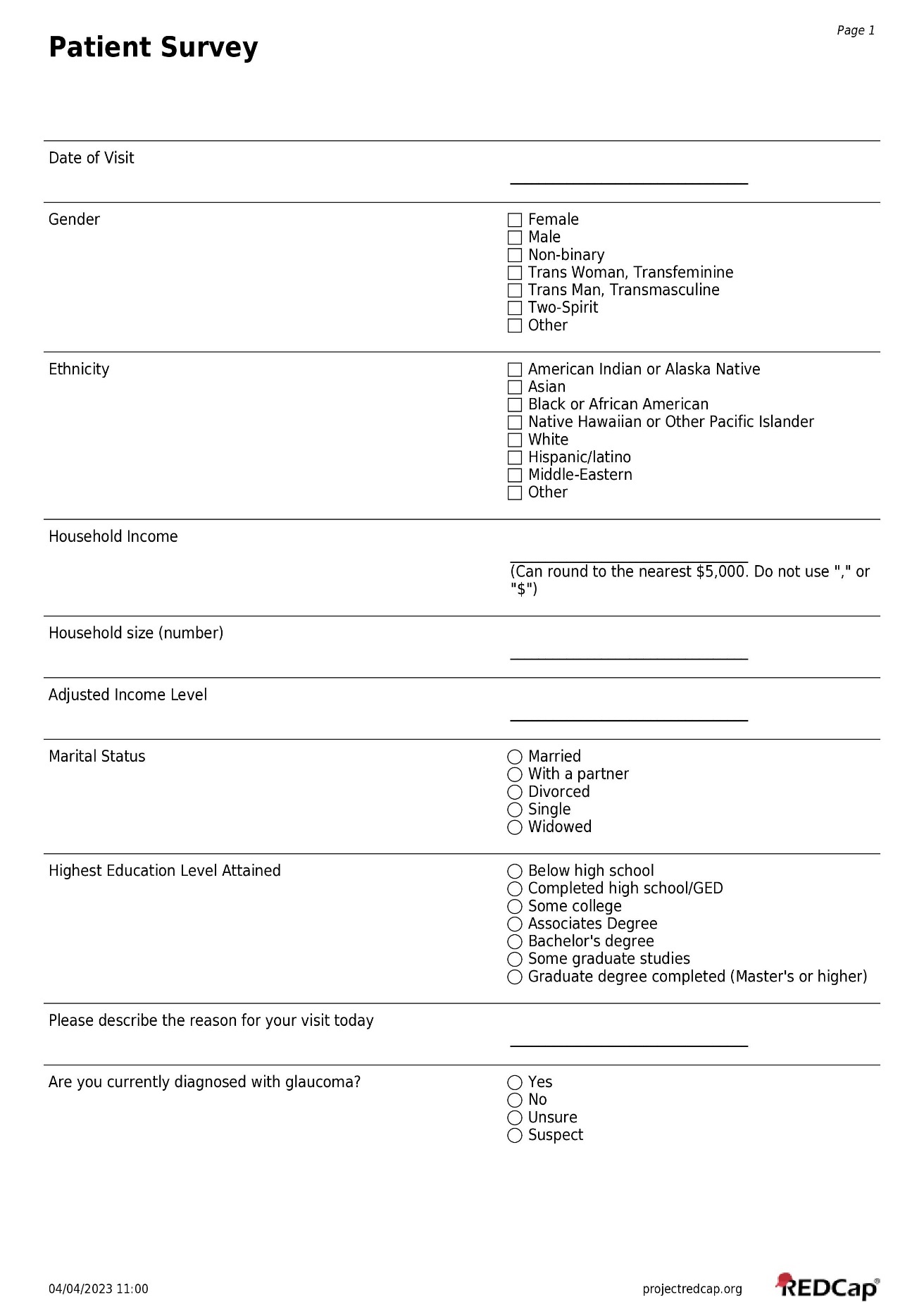


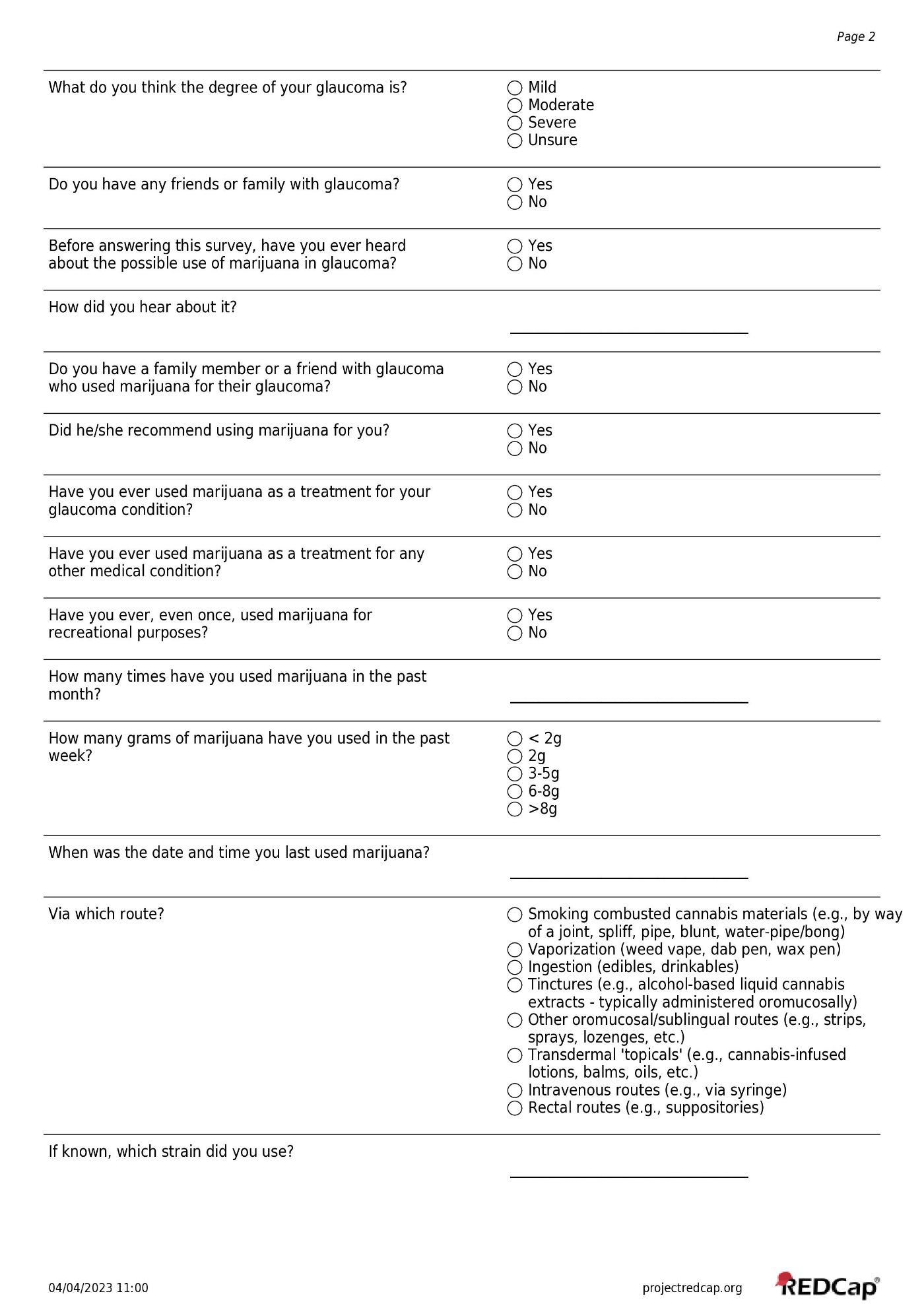


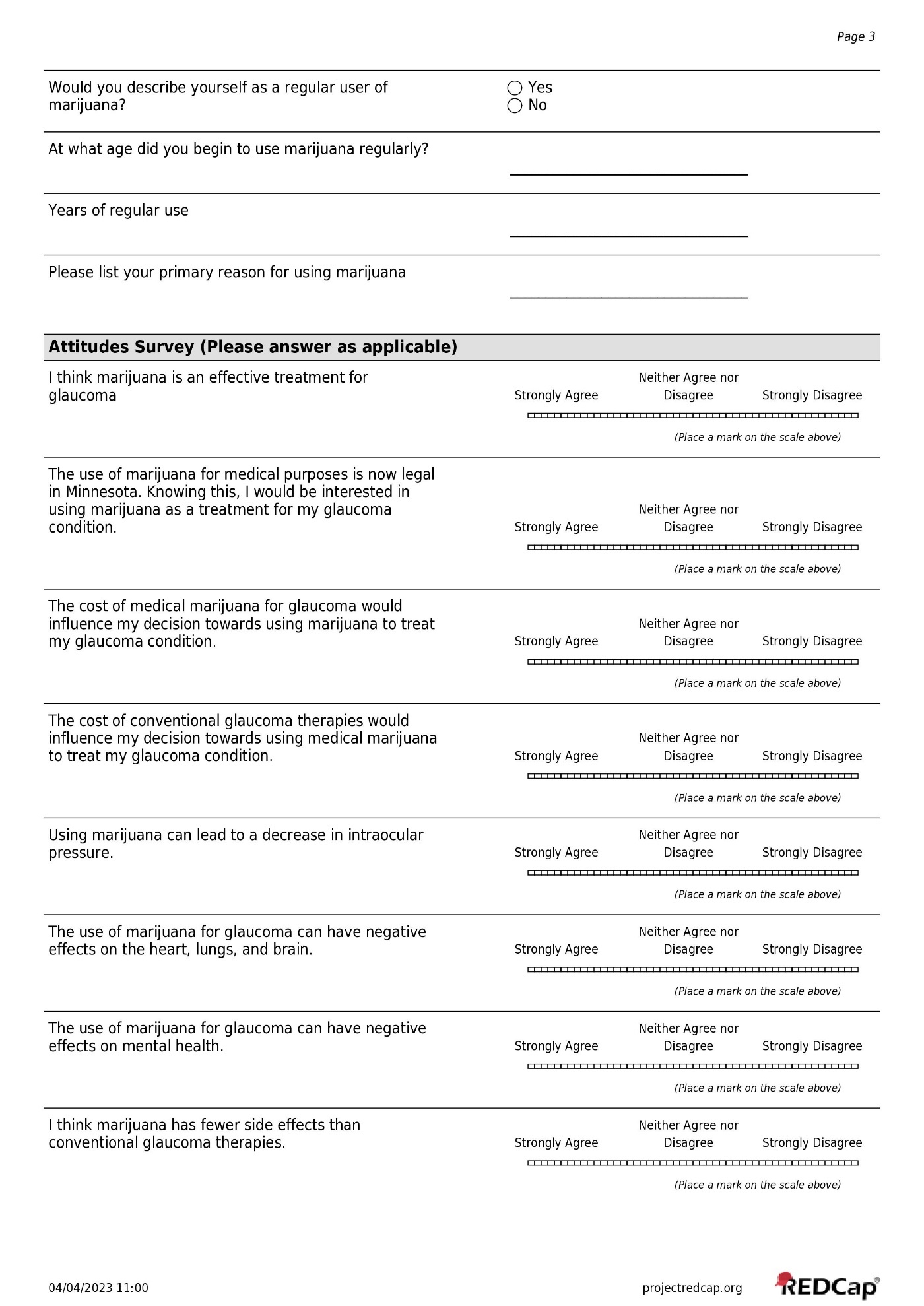

Supplement: Supplementary file 1 — Supplementary file1 (DOCX 1.58 MB) [file 10792_2025_3846_MOESM1_ESM.docx]
