# Supplementary material for: An assessment of the prevalence of cannabis use in eye clinic patients and its implications on glaucoma diagnosis and management
Source: Int Ophthalmol. 2025 Nov 16;45(1):484. doi: 10.1007/s10792-025-03846-2 (PMC12620322; doi:10.1007/s10792-025-03846-2)
Supplement: Supplementary file 2 — Supplementary file1 (DOCX 16 kb) [file 10792_2025_3846_MOESM2_ESM.docx]

**Online resource 2.** Attitudes Survey.

Attitudes Survey: Please answer as applicable.

(Response options: Strongly agree, agree, not sure/no opinion, disagree, strongly disagree)

| 1. I think marijuana is an effective treatment for glaucoma |
| --- |
| 1. The use of marijuana for medical purposes is now legal in Minnesota. Knowing this, I would be interested in using marijuana as a treatment for my glaucoma condition. |
| 1. The cost of medical marijuana for glaucoma would influence my decision towards using marijuana to treat my glaucoma condition. |
| 1. The cost of conventional glaucoma therapies would influence my decision towards using medical marijuana to treat my glaucoma condition. |
| 1. Using marijuana can lead to a decrease in intraocular pressure. |
| 1. The use of marijuana for glaucoma can have negative effects on the heart, lungs, and brain. |
| 1. The use of marijuana for glaucoma can have negative effects on mental health. |
| 1. I think marijuana has fewer side effects than conventional glaucoma therapies. |
| 1. I believe marijuana is a safe alternative to glaucoma medications. |
| 1. I believe marijuana is a safe alternative to glaucoma surgery. |
| 1. I believe marijuana use for medical purposes, including glaucoma, should be legal in all states. |
| 1. I believe marijuana use for recreational purposes should be legal in all states. |
| 1. I believe the use of marijuana to treat glaucoma is more effective than other glaucoma treatment options. |
| 1. I think using marijuana can help prevent glaucoma. |
| 1. I would be interested in using marijuana for my glaucoma condition even if it is less effective than my regular glaucoma medications. |
| 1. I would be interested in using marijuana for my glaucoma condition even if it costs more than my regular glaucoma medications. |
| 1. I have trouble paying for my current glaucoma medications. |
| 1. I am satisfied with the way my doctor is treating my glaucoma condition. |
| 1. I am satisfied with my current glaucoma medications. |
| 1. I believe my current glaucoma medications effectively control my glaucoma condition. |
| 1. If my doctor won't prescribe medical marijuana for my glaucoma, I will seek other doctors who will. |
